# Solid Phase Extraction for Monitoring of Occupational Exposure to Cr (III)

**Published:** 2007-12-11

**Authors:** S.J. Shahtaheri, M. Khadem, F. Golbabaei, A. Rahimi-Froushani

**Affiliations:** 1 Department of Occupational Health, Tehran University of Medical Sciences, Iran.; 2 Department of Epidemiology and Biostatistics, Tehran University of Medical Sciences, Iran.

**Keywords:** chromium, sample preparation, atomic absorption spectrometery

## Abstract

Chromium is an important constituent widely used in different industrial processes for production of various synthetic materials. For evaluation of workers’ exposure to trace toxic metal of Cr (III), environmental and biological monitoring are essential processes, in which, preparation of samples is one of the most time-consuming and error-prone aspects prior to analysis. The use of solid-phase extraction (SPE) has grown and is a fertile technique of sample preparation as it provides better results than those produced by liquid-liquid extraction (LLE). SPE using mini columns filled with XAD-4 resin was optimized regarding to sample pH, ligand concentration, loading flow rate, elution solvent, sample volume, elution volume, amount of resins, and sample matrix interferences. Chromium was retained on solid sorbent and was eluted with 2 M HNO_3_ followed by simple determination of analytes by using flame atomic absorption spectrometery. Obtained recoveries of metal ion were more than 92%. The optimized procedure was also validated with three different pools of spiked urine samples and showed a good reproducibility over six consecutive days as well as six within-day experiments. Through this study, suitable results were obtained for relative standard deviation, therefore, it is concluded that, this optimized method can be considered to be successful in simplifying sample preparation for trace residue analysis of Cr in different matrices for evaluation of occupational and environmental exposures. To evaluate occupational exposure to chromium, 16 urine samples were taken, prepared, and analyzed based on optimized procedure.

## Introduction

Heavy metals can be considered as a unique class of environmental toxicant. They occur and persist in nature and most of them are advantageous to humans because of their vast usages in different industries, agriculture, and medicine. However, they may pose health hazards to the public because of their presence in air, water, food chains as well as to the workers engaged in mining, smelting, alloy, painting, electroplating, pesticides, and the variety of industrial activities. Some heavy metals such as chromium have a wide range of toxicities, leading to toxic effects on the renal, respiratory, and nervous systems. Also, chromium (VI) compounds are classified as A1 confirmed human carcinogens by ACGIH.

The water soluble hexavalent chromium compounds are highly irritants of nasopharynx, larynx, lungs, and skin. Cr (VI) compounds have been implicated as reparable for perforated nasal septa, rhinitis, nosebleed, kidney damage, and skin ulceration. In body fluids, Cr (VI) is reduced to Cr (III), thus, determination of Cr (III) in these fluids is useful where the workers exposure are just taken placed to Cr (VI) (Frank, 1996; Hathway et al. 1996; WHO, 1998; Bingham et al. 2004). However, in recent years, some studies have been performed on different environmental samples, including various wastewater samples (Narin et al. 2006; Baytak et al. 2006; Bulut et al. 2007; Suvardham et al. 2007), electroplating wastewater (Yalcin and Apak, 2006) and bottled mineral water as well as artificial seawater (Soylak, 2004).

Because, usages of this heavy metal are unavoidable, from the occupational viewpoints, study of this compound is of great interest. One of the most important aspects of metal studies is determination of such compounds in different matrices. In biological samples, either exposed compounds or their metabolites, metals are mostly present at trace level, causing major problems in their determination stages (McDowall, 1989; Shahtaheri et al. 1998; Shahtaheri et al. 2005). Therefore, an essential need for precise, reliable, and sensitive techniques for the analysis of such trace chemicals in biological samples has been clearly recognized (Poole, 1990; Hennion and Scribe, 1993; Maria, 2000).

Although the use of detection system has improved the selectivity of the analytical procedures, these sensitive and selective methods required expensive equipments; moreover, they may not be available in most laboratories. Consequently, sample preparation procedures which can be performed in any laboratory have been developed to simplify analytical approaches as these reduced expenses too (Mc Dowall, 1989; Poole 1990; Maria, 2000; Shahtaheri and Stevenson, 2001). For this purpose, to extract heavy metals, many sample preparation procedures are being used such as Soxhlet extraction (Mitra, 2003), liquid liquid extraction (LLE) (Ibrahim and Suffet, 1988; Tuzen, et al. 2002; Bouabdallah et al. 2006), supercritical fluid extraction (SFE) (Takeshita et al. 1999), and solid phase extraction (SPE) (Ramesh et al. 2002; Akman et al. 2002; Tuzen et al. 2004; Tokman and Akman, 2004), in which, Soxhlet and LLE are time consuming procedures and also the recoveries obtained from such methods are not reproducible and efficient. Therefore, more sensitive and precise methods are required to measure trace heavy metals in biological and environmental samples. In contrast, solid phase extraction methods using silica has proven useful in simplifying sample preparation prior to analytical technique. This method refers to the adsorption of chemical constituent from a liquid sample (water, urine, *etc.*) on a solid sorbent and subsequent desorption of retained constituent by elution from the sorbent. Through this procedure, isolation and purification of the compound of interest can be achieved in a short time and only low volumes of solvents are used during the application of the method. The use of commercially available low-cost vacuum manifolds allows many samples to be proceed simultaneously. Furthermore complete automation of procedures based on SPE is now possible using commercially available instrumentation (Sturgeon et al. 1980; Cesur, 2003; Soylak and Dogan, 2003; Narin et al. 2004; Focant et al. 2004; Petterson et al. 2004). A wide range of phases based on silicas are also available from many suppliers, including reversed phase, normal phase, ion exchange, and mixed mode phases. These phases can be screened and selected, depending on the chemical nature of the analyte (Hennion, 1999). Therefore, the variety of available phases can improve the selectivity of the sample preparation procedures.

This study was aimed to achieve optimum factors necessary for development of an optimized procedure for chromium (III) present in water and urine samples, leading to a simple protocol of SPE method.

## Material and Methods

### Chemicals and reagents

All solutions were prepared using distilled water. chromium stock solution was prepared from appropriate amount of the nitrate salt of this analyte (Merck, Darmstadt, Germany) as 1000 mg/lit solution in 0.01 M HNO_3_. Working and standard solutions were prepared daily by dilution of the stock solution. Acids and other chemicals used in this study were obtained from Merck, Darmstadt, Germany. Standard buffered solution at various pH values, ammonium pirrolidine dithio carbamate (APDC), amberlite XAD-4 resin (20–40 mesh) were also purchased from Merck, Germany.

### Apparatus

Determination of chromium was with spectra AA/plus 20, Varian flame atomic absorption spectrometer (FAAS; Varian, Australia) using air-acetylene flame. The operating parameters for metal of interest were set as recommended by the manufacturer. The pH values of the solutions were measured by a digital pH meter model Metrohm 744. Amount of reagents were measured using a Satorius CP225D balance (Sartorius, Germany).

### Preparation of mini columns

Glass mini columns (100 × 10 mm) were packed with 500 mg resin. After packing, a little amount of glass wool was placed at both ends of the glass tube. Before using the column, XAD-4 resin was washed by methanol, water, 1 M HNO_3_, water, 1 M NaOH, and water, respectively. Finally, resin was pre-concentrated with buffer solution.

### Pre-concentration procedure

In this study, SPE using amberlitr XAD-4 resin was optimized with regard to sample pH, sample and eluent flow rates, elution solvent, eluent volume, ligand concentration, amount of resin, and sample volume. Fifty milliliter solution containing 20 μg of Cr (III), 10 ml buffer solution with desired pH and 6 ml APDC solution were prepared. Samples were then passed through the column packed in our laboratory at a flow rate of 5 ml/min. The column was then washed with 5–10 ml of the same buffer solution. Therefore, the metal ions were eluted from the mini column with 10–15 ml of different solvents. Finally, the concentration of chromium in the solutions, including standards, spiked, and real samples were determined by FAAS.

## Results

### Effect of sample pH

The influence of sample pH on adsorption of Cr (III) ion on XAD-4 resin was investigated, using different pH values of 2, 4, 7, and 9. The pH values were adjusted by buffer solution. Fifty milliliter of sample containing 20 μg of Cr (III) and 6 ml APDC solution was loaded on the column. The column was then washed and the retained analyte was eluted using 2 M HNO_3_. Table 1 shows the influence of sample pH on extraction recovery for Cr (III). Finally, the sample pH of 9 was selected as an optimum value for further experiments.

### Effect of APDC concentration

The concentration of Ammonium Pirrolodine Dithio Carbamate (APDC) is one of the important parameter could affect on recovery obtained from the optimized method. Through this investigation, the amount of 0.01–0.07% (w/v) of APDC were used. The results obtained from this investigation showed that, by increasing APDC concentration up to 0.05%, the recoveries are also increased, afterward, constant values are recovered.

### Effect of eluent type

Evaluation of eluent strength onrecovery of Cr (III) was another experiment performed during this study. Five solvents were screened for their ability to produce optimum elution of the retained Cr (III) from the XAD-4 resin. They were 1 M HCl, acetone, 1 M HNO_3_ in acetone, 1 M HNO_3_, and 2 M HNO_3_. The same sequence of conditioning, washing, and elution were used as in the previous section. The results are presented in Table 1. A quantitative recovery (98%) was obtained for Cr (III) ion, using 2 M HNO_3_ as an efficient eluent and, therefore, it was used as a suitable solvent for further studies.

### Effect of eluent volume

Eluent volume is an influencing parameter, affecting on the pre-concentration of analytes using SPE. Enrichment of the analyte in SPE is achieved by applying large volume of sample and eluting the analyte in a minimum volume of eluent. The volume of the eluent must be just sufficient to elute the compound of interest from the sorbent. Thus, the recovery of metal ion was studied in applying different eluent volumes of 5, 10, 15, and 20 ml. The results are given in Table 1. Volumes of 15 and 20 ml provided efficient recovery for the analyte of interest. In order to obtain confident concentration factor, the smallest satisfactory volume (15 ml) was chosen for the next experiments.

### Effect of eluent flow rate

In order to evaluate the influence of eluent the eluent flow rate on recovery of the analytes, retained metal ion was eluted, using eluent at different flow rates of 2, 5, 7, and 10 ml/min. The same sequence of conditioning, washing, and elution were used as in the previous section. As the Table 2 shows, metal of interest was quantitavely recovered in eluent flow rate up to 7 ml/min. Flow rate of 5 ml/min was then selected as an optimum value for the next experiments.

### Effect of sample volume

In order to evaluate the sample volume, 20 μg of Cr (III) was diluted into different volumes of 50, 150, 250, 500, and 750 ml. These samples loaded on XAD-4 mini columns. The columns were then washed and the retained analyte was eluted according to the optimized method .The results have been shown in Table 2. It can be seen that up to 500 ml of samples could be applied without significant loss of recovery (94%). Therefore, the highest concentration factor was 33.3% when the final volume was 15 ml.

### Effect of sample flow rate

Following a demonstration of the feasibility of using large sample volumes, the effect of sample flow rate on metal ion adsorption on XAD-4 was studied in different sample flow rate of 2, 5, 7, and 9 ml/min. Fifty ml sample, using optimum pH, containing 20 μg of metal ion and APDC solution were prepared. Thereafter, the same sequence of conditioning, washing, and elution were used as in the previous section. No significant reduction in recovery was found for sample flow rate up to 7 ml/min. Flow rate of 5 ml/min as an appropriate value was then used to continue further experiments. Table 2 shows the results obtained from this experiment.

### Effect of XAD-4 sorbent mass

The effect of XAD-4 amount was investigated, using 100 and 500 mg sorbent packed in a mini column. The same sequence of preparation procedure was used as in the previous section. The obtained recovery of metal ion was more efficient when 500 mg was utilized (see Table 2).

### Effect of matrix

The effect of various matrix possible ions, mostly present in the environmental and biological samples, including Na^+^, K^+^, Mg^2+^, Ca^2+^, and SO4^2−^ was another parameter, influencing the efficiencies of analyte recoveries. The procedure was performed, using 50 ml sample containing 20 μg of analyte and different concentration of matrix ions. The results have been shown in Table 3.

### Reproducibility

As spiked urine may contain some interference compounds similar to the real sample, it can be considered as an appropriate sample, preferably better than water sample for validation of the optimized method. Therefore, further experiments were carried out in urine; however, the working samples were made in aquatic solution. A preliminary validation of the possible use of the optimized method for measuring metal ion of chromium (III) in urine was carried out, using spiked samples. Samples of 50 ml were used for extraction with subsequent FAAS. Linear standard curves (extracted) over the concentration range of 1, 1.5, and 2 μg/ml were obtained each day (n = 6) for six consecutive days with a correlation coefficient of 0.995 or greater. The day-to-day (for six consecutive days) and within-day reproducibility of the method was investigated. Table 4 shows the results obtained from this experiment.

### Chromium in urine

Finally, real samples of urine were obtained from 16 workers employed in the relevant industries, using the optimized method. The results obtained from this experiment have been shown in Table 5.

## Discussion

The results showed that efficient recovery was obtained from XAD-4 resin using sample pH of 9. For Chromium (III), the amount of the analyte recovered from sorbent at sample pH values of 2, 4, and 7 was also efficient. However, the pH value of the sample should be adjusted according to the chemistry of the compound of interest. It seems that, at sample pH of 9, the analyte of interest mostly is in the ionized form, making it to be easily retained on the ionized ligand already conjugated to the sorbents. From these pH values, sample pH of 9 was selected for further study as this pH seems to be rather confident value. In this study, a non polar sorbent was used, in which, there was no affinity between this type of sorbent and the ionized analyte, so, there was a need of conjugating ionized ligand on the sorbents to follow up an ionized extraction mechanism. APDC showed to be an appropriate ligand for capturing chromium (III) from the sample, however, from the four concentrations of the ligand, the amount of 0.05, and 0.07 (%) showed to be good enough for efficient retaining of the analyte. However, for preventing saturation of the sorbent with the ligand and also reduce the reagents through extraction process, the lesser percentage of the ligand (0.05%) was used as this amount provides the same recovery needed for the method.

Understanding the chemistry of the compound under analysis such as ionizability and hydrophobicity can be useful in designing appropriate conditions to obtain efficient extraction recovery. Highly ionic compounds can result in a strongly retained analyte making elution difficult and leading to subsequent poor recovery from ionic conjugated sorbrnt. From the eluents used in this study, as the Table 1 shows, the HNO_3_ based solutions were more efficient and from these solvents, 2 M HNO_3_ was selected, because, it was organic free eluent and can prevent co-elution of organic compounds possibly present in the real samples as well as reducing exposure to such evaporative and hazardous compounds. Moreover, maximum recovery has been ahieved using this eluent. The results obtained from an evaluation of the elution volume (Table 1) showed that the smallest satisfactory volume for 2 M HNO_3_ from XAD-4 sorbent was 15 ml. As a consequence, the volume required to elute the analyte from the sorbent, depends on two important parameters. First, the strength of its retention, a solvent with greater elution strength can be used to elute an analyte in less volume, but may incorporate undesirable contaminants into the eluted fractions; secondly, the sorbent mass used in SPE, in which, using a larger sorbent mass cartridges requires an increase elution volume to be applied. As it can be seen in Table 1, the lowest satisfactory eluent volume is 15 ml, giving a suitable concentration factor of 33.3. Using this volume, efficient recovery of 96.92% can be achieved. Although the low eluent volume caused to achieve an appropriate concentration factor, however, the faster elution of 15 ml eluent by itself can affect on the whole analysis time when numerous samples is going to be applied. Therefore, through this experiment, the reduced eluent flow rate of 5 ml/min was enough to reduce the elution time to one third. The experiment on sample volume allowed an accurate measurement as low as 40 μg/ml (0.04 ppm) of chromium when a large sample volume (500 ml) is applied on the column, resulting in a possible trace enrichment of the analyte with an appropriate concentration factor of 33.3 which was compatible to the current atomic absorption spectrometry detection system. As the high volume of sample is applicable with an efficient recovery, it would be of favorite if high sample flow rate can be applied. In this study sample flow rate of up to 9 ml/min were applied with acceptable recovery of 92% and more (see Table 2). Therefore, to be confident, the sample flow rate of 5 ml/min was selected, providing a reduced extraction time for as large as 500 ml sample volume. However, as the results shows, it would be possible to increase the sample flow rate even more as 9 ml without significant loss in the analyte recovery.

Regarding the sorbent mass, it was seen that, 100 mg sorbent was not appropriate amount as breakthrough was happened through the experiment, so, a non efficient amount of 68% of the retained compound was recovered which is not acceptable in our optimized method. By using the sorbent mass as large as 500 mg, it allowed that a longer interaction to be taken place, causing retention of significant amount of chromium on the sorbent and subsequent efficient recovery of 98%. However, using large amount of sorbent mass needs a large volume of washing solvent and eluent to be applied for the efficient removal of possible interferences. In order to show effect of possible matrix components on the optimized method, the similar ions illustrated in Table 3, having three different concentrations were added to the sample. The ions added to the samples are mostly present in the real environmental samples and can be used as closely related interferences present in matrices. The results clearly showing the non-effectiveness of the all of added components for each concentration on the recoveries obtained from optimized method. As it can be seen, the recoveries are 92% or greater which is promising either no cross-reactivity is taken place between added interferences and the XAD-4 or no co-elution is happened.

In order to validate the method, reproducibility of the optimized method was performed for day-to-day and within-day experiments. A linear standard curve (for extracted sample) over the range concentrations of 1, 1.5, and 2 μg/ml was obtained every day for 6 consecutive days (n = 6) with the correlation coefficient of 0.95 or grater. In within-day experiments evaluation, six experiments were performed per day for three consecutive days. The extraction procedure was reliable and reproducible from day-to-day and within-day. Coefficient of variations (CV%) of 0.830, 1.01, and 1.14 were obtained for 1, 1.5, and 2 μg/ml respectively for day-to-day and 2.01, 2.04, and 0.272 at the same concentrations respectively for within-day, showing suitable accuracy and precision (see Table 4). Finally, an assessment on occupational exposure to chromium also showed a proper applicability of optimized method in occupational exposure. The results illustrated overexposure of some workers to chromium (see Table 5).

Comparatively, the method recently reported (Bouabdallah et al. 2006) has used liquid liquid extraction (LLE) for some heavy metals. Although the technique may be useful in some conditions, however, there are still no basic rules for selection of a solvent system for extraction of given analyte, therefore, selection of a solvent is still empirical and of course time consuming step as well as a tedious stage. Sometimes, emulsion formation of the sample makes the analyte extraction too hard as such solutions are extremely difficult to be broken and often cannot be isolated by either centrifugation or ultra-sonication. Other problems associated with LLE include: the use of large volumes of toxic and sometimes inflammable solvents, contamination of extracts from solvents and glassware, low recovery due to degradation by heat, and volatilization or adsorption to glassware. Therefore, due to such problems, nowadays, there is a strong trend towards replacing LLE by SPE. Based on reported methods (Tuzen et al. 2002; Sturgeon et al. 1980; Soylak and Dogan 2003; Narin et al. 2001; Hennion 1999), for optimizing SPE, authors generally have used 5–6 factors to optimize the method for environmental samples (Bulut et al. 2007; Narin et al. 2006; Baytak et al. 2006), while, in this study, 9 parameters were screened, including significant factors of sorbent mass, eluent flow rate, sample matrix interferences, and also ligand concentrations. This allows that a robust and more reliable method is introduced for biological monitoring of Cr (III). Therfore, to make an advantage from this study compare to the other studies (Ramesh et al. 2002; Akman et al. 2002; Tuzen et al. 2002; Tokman and Akman 2004; Sturgeon et al. 1980; Soylak and Dogan 2003; Narin et al. 2001), further experiments of reproducibility of the method were carried out on spiked urine samples to validate the possible use of the optimized SPE for measuring Cr (III) when an environmental study and biological monitoring of worker exposed to such pollutant are required. Although the concentration factor obtained from this study is high, however, the relatively low sensitivity of the AAS did not allowed the authors to get even more concentration factor.

## Conclusions

Through this study factors influencing SPE were optimized, showing an efficient sample preparation procedure for chromium (III) as a solid phase extraction method has more advantages than liquid liquid extraction. Depending on the chemical and physical properties of the analyte, manipulating factors including sample pH, ligand concentration (APDC), loading flow rate, elution solvent, sample volume (up to 500 ml), elution volume, amount of resin (XAD-4), and sample matrix interferences can play essential roles in optimizing the method, providing reliable, easy to use, and cost effective procedure to overcome difficulties associated with other sample preparation techniques. The concentration factor was 33.3 and the resin can be used several times. The optimized method was also used for pre-concentration of urinary Cr (VI) in relevant industry and promising some other applications when analysis of trace heavy metals in biological and environmental samples is of interests. The authors are sure that, SPE is a highly fertile area for sample preparation method and based on the needs and facilities, these method protocols can be further developed in the near future.

## Figures and Tables

**Table 1:** Effect of pH, Ligand Concentration, eluent type, and eluent volume on recovery of Cr (III) on XAD-4 resin (eluent: 2 M HNO_3_).

**pH**	**Mean(%) ± SD (N = 5)**	**Ligand concentration [w/v(%)]**	**Mean(%) ± SD (N = 5)**	**Eluent type**	**Mean(%) ± SD (N = 5)**	**Eluent volume (ml)**	**Mean(%) ± SD (N = 5)**
				1M HCL	14 ± 5.47		
2	82 ± 4.47	0.01	52 ± 4.47			5	40.00 ± 7.07
				Acetone	74 ± 8.94		
4	86 ± 5.47	0.03	74 ± 5.47			10	63.00 ± 4.47
				(HNO_3_ in Acetone)	98 ± 4.47		
7	96 ± 5.47	0.05	98 ± 4.47			15	96.92 ± 4.21
				1M HNO_3_	96 ± 5.47		
9	98 ± 4.47	0.07	98 ± 4.47			20	98.00 ± 4.47
				2M HNO_3_	98 ± 4.47		

**Table 2:** Effect of eluent flow rate, sample volume, sample flow rate, and sorbent mass on recovery of Cr (III) from XAD-4 resin (eluent: 2 M HNO_3_).

**Eluent flow rate (ml/min)**	**Mean (%) ± SD (N = 5)**	**Sample volume (ml)**	**Mean (%) ± SD (N = 5)**	**Sample flow rate (ml/min)**	**Mean (%) ± SD (N = 5)**	**Sorbent mass (mg)**	**Mean (%) ± SD (N = 5)**
		50	98 ± 4.47				
2	96 ± 5.47			2	100 ± 0.00		
		150	98 ± 4.47			100	68 ± 4.47
5	796 ± 5.47			5	94 ± 5.47		
		250	96 ± 5.47				
7	94 ± 5.47			7	94 ± 5.47		
		500	94 ± 5.47			500	98 ± 4.47
10	90 ± 0.00			9	92 ± 4.47		
		750	44 ± 8.94				

**Table 3:** Effect of matrix ions on recovery of Cr (III) from XAD-4 resin (eluent: 2 M HNO_3_).

**Ions (added)**	**Concentration (g/l)**	**Recovery (%)**	
		**Mean ± SD, N = 5**
Na^+^(NaCl)	2.5	98 ± 4.47
	10	102 ± 2.37
	20	96 ± 4.47
K^+^(KCl)	0.3	98 ± 5.47
	0.5	96 ± 4.47
	1	92 ± 4.47
Mg^2+^(MgCl_2_)	0.3	98 ± 5.47
	0.5	98 ± 5.47
	1	94 ± 5.47
Ca^2+^(CaCl_2_)	0.3	98 ± 4.47
	0.5	100 ± 0.00
	1	94 ± 5.47
SO_4_^2−^ [(NH_4_)_2_SO_4_]	0.5	98 ± 5.57
	1	96 ± 5.47
	1.5	94 ± 4.47

**Table 4:** Day-to-day (D-day) and within day (W-day) reproducibility of Cr (III) spiked in urine, sample volume: 50 ml, N = 6.

**Statistical data**	**Concentration added (μg/l)**					
	**1**		**1.5**		**2**	

	**D-day**	**W-day**	**D-day**	**W-day**	**D-day**	**W-day**
Mean	0.976	0.970	1.452	1.440	1.920	1.940
SD	0.816	1.952	0.983	1.095	1.095	1.264
CV%	0.830	2.01	1.01	2.04	1.14	0.272

**Table 5:** Urinary chromium (III) detected in samples taken from workers employing in relevant industries based on optimized procedure.

**Sample No.**	**Cr (III) Conc. (ppb)**
1	5.6
2	ND*
3	0.59
4	7.71
5	1.67
6	14.56
7	8.84
8	ND
9	25.57
10	ND
11	48.37
12	14.73
13	24.25
14	35.56
15	16.82
16	38.96

Mean ± SD	19.45 ± 15.30

BEL**	34.80

* Not Detectable.

** Biological Exposure Level.
